# Determination of Risk Factors Associated with the Failure of 12 Weeks of Direct-Acting Antiviral Therapy in Patients with Hepatitis C: A Prospective Study

**DOI:** 10.1155/2022/6054677

**Published:** 2022-05-06

**Authors:** Phuong Nguyen Thi Thu, Mai Ngo Thi Quynh, Linh Pham Van, Hung Nguyen Van, Hoi Nguyen Thanh

**Affiliations:** ^1^Hai Phong University of Medicine and Pharmacy, Vietnam 18000; ^2^Hai Phong International Hospital, Vietnam 18000

## Abstract

**Introduction:**

Direct-acting antivirals (DAAs) have significantly improved the efficacy and tolerability of the treatment of hepatitis C virus (HCV). However, studies conducted on actual patients with the aim of predicting the risk associated with treatment failure are lacking.

**Methods:**

Our study enrolled 334 new HCV patients and assessed the effectiveness of treatment and predicted the risk of failure to achieve sustained virological response (SVR) by developing a multiple logistic model. Our study compared the variables between the two groups, those who did (group 0, *n* = 239) and did not achieve SVR (group 1, *n* = 95).

**Results:**

The cure rate of HCV at 12^th^ week in our study was 71.56%. We found that advanced cirrhosis, HCV genotype, HBV coinfection, rapid virological response (RVR), fibrosis index (FIB-4) score, serum levels of AST, ALP, hemoglobin, and viral load before treatment were prognostic factors associated with rate of failure to achieve SVR at week 12 of DAA therapy. In the multiple logistic model, eight significant predictors including advanced cirrhosis status, HCV genotype, RVR, AST/ALP levels, FIB-4 score, and viral load before treatment predicted the risk of failure with excellent model performance (area under the receiver operating characteristic curve (AUC_ROC_) [95% CI] =0.986 (0.971-0.999)). RVR and advanced cirrhosis were the two strongest predictors with odd ratios (95% CI) =9.72 (2.8, 39.28) and 51.54 (6.39, 139.82), respectively.

**Conclusion:**

The multiple logistic regression model included significant factors to estimate the probability of failure to achieve SVR, which could improve HCV treatment strategy.

## 1. Introduction

Chronic hepatitis C virus (HCV) infection continues to be a serious global health problem, with 71 million people infected with HCV [1]. To date, HCV remains the leading cause of chronic liver disease and hepatocellular carcinoma (HCC) worldwide in both developed and developing countries [2]. In Vietnam, the HCV is estimated to affect approximately 1%-2% of the total population [3, 4]. It should be noted that the distribution of HCV genotypes and the clinical manifestations of HCV in Southeast Asian countries such as Vietnam are different from those in Europe and America [5]. The hepatitis C virus is transmitted through unsafe medical and nonmedical injections, unscreened blood and blood products, and sharing of injection equipment, to name just a few routes. This is common in developing and low-middle income countries in Southeast Asia, which is likely responsible for the higher prevalence of HCV there [1]. To date, HCV has been classified into seven recognized genotypes including genotype 1, genotype 2, genotype 3, genotype 4, genotype 5, genotype 6, and mixed genotype [1, 6]. HCV genotype 6 accounts for only 2% of clinical manifestations in countries worldwide but is very common in Southeast Asian countries including Vietnam [5, 7].

The goal of HCV treatment is sustained virological response (SVR), defined as the absence of detectable viral RNA levels in the blood 24 weeks after completion of treatment. Besides the goal of eradicating the virus, HCV treatment also needs to prevent the development of cirrhosis and its complications. Global efforts to improve the efficacy and tolerability of HCV treatment, including an understanding of the HCV genome, have paved the way for the development of many direct-acting antivirals (DAAs) [8]. Sofosbuvir and velpatasvir demonstrated clinical benefits in a small number of patients with HCV genotypes 1, 2, 3, 4, 5, and 6 in a phase II/III study. However, studies beyond clinical trials, involving patients in actual clinical settings, are needed to confirm their clinical efficacy [9, 10]. Although DAA has been discovered and used in the early years of the twenty-first century in other parts of the world, in Vietnam, the Ministry of Health has only recently (2019) implemented a new list of health insurance drugs, in which the approved DAA regimen includes the following: (1) sofosbuvir and ledipasvir (SOF/LDV), (2) sofosbuvir and velpatasvir (SOF/VEL), and (3) sofosbuvir and daclatasvir [11].

Predicting the success rate of achieving SVR at weeks 12 and 24 of DAA therapy can help guide treatment plans as well as HCV prognosis. Undetectable HCV RNA 4 weeks after the start of treatment was also considered a predictor, defined as rapid virological response (RVR) [12]. The strongest SVR predictors for HCV in general were genetic polymorphisms in IL28B, HCV genotype, fibrosis stage, and RVR [12]. Other predictors of response to HCV treatment include patient characteristics (e.g., age, BMI, insulin resistance, and sex), pretreatment HCV RNA levels, coinfections, dose regimen and duration of treatment, virological response during treatment, and adherence. A model to predict HCV treatment failure after initiating DAA therapy has been developed that includes variables for age, sex, body mass index (BMI), population ethnicity, insurance type, HCV genotype 1, HCV treatment history, fibrosis stage, Child-Turcotte classification in patients with cirrhosis, hepatitis B virus and/or HIV coinfection, history of solid organ transplantation, dialysis status, diabetes or comorbid mental illness, and the presence of drug-drug interactions (DDI) with the expected DAA regimen in 1,253 patients [13].

We performed a prospective study in treatment DAA-naïve patients with chronic hepatitis C to predict the risk of failure to achieve SVR at week 12 of DAA therapy by developing a multiple logistic model based on patient factors, preclinical characteristics before treatment, and genotype of HCV virus in Vietnamese HCV patients.

## 2. Materials and Methods

### 2.1. Study Design

The observational study was prospectively designed with a weekly follow-up after the initiation of DAA therapy for 12 weeks. The study was conducted at the Hai Phong International General Hospital from December 2019 to July 2021. Research data were collected from electronic medical records and medical examination processes of specialists. Patients were examined, checked for compliance with drug use, and underwent a blood test every four weeks. Treatment failure was determined by the absence of SVR at 12 weeks (SVR 12) after treatment completion.

### 2.2. Patients

Our study selected HCV patients from the Gastroenterology and Hepatobiliary Clinic of Hai Phong International General Hospital from January 2019 to July 2021 using the following inclusion criteria: age>18 years, DAA-naïve patients with HCV infection, and indicated for treatment with DAAs. Patients with current hepatocellular carcinoma or renal failure with an estimated glomerular filtration rate (eGFR) of <30 mL/min/1.73 m^2^ were excluded from the study.

HCV infection was diagnosed using two methods [14]: an anti-HCV antibody test to screen for HCV infection and a subsequent confirmatory nucleic acid test for HCV ribonucleic acid (RNA).

### 2.3. Control Variables

Demographic and baseline characteristics of the study patients, including age, sex, weight, and height, were collected at the time of treatment initiation. The co-diseases were determined according to the International Classification of Disease (ICD) – 10 code.

The fibrosis index based on four factors (FIB-4) is calculated [15]:

(1) $$\begin{array}{c} \text{FIB}-4=\frac{\left(\text{age x AST}\right)}{\text{Platelet count x }\sqrt{\text{ALT}}}. \end{array}$$

The FIB-4 upper limit of normal (ULN) was 34 U/L for females and 36 U/L for males.

Estimated eGFR is estimated using the following formula [16]:

(2) $$\begin{array}{c} eGFR\;\left(mL/\min\right)=\left[\left(140-age\right)\times Wt/\left(0.814\times S.Cr\;\text{in }µmol/L\right)\right]\times \left(0.85\;\text{if }female\right)\left[eGFR\;must\;be\;corrected\;\text{for }surface\;area\right], \end{array}$$

where *Wt* = weight and *S*.*Cr* = serum creatinine.

The HCV viral load was measured using Roche COBAS AmpliPrep/COBAS TaqMan, Version 2 (Roche, Pleasanton, CA, USA) according to the manufacturer's instructions with a lower limit of quantification and detection of 15 IU/mL before the start (baseline viral load) and at 12th week of DAA therapy.

For HCV genotyping, viral RNA from HCV RNA-positive samples were reverse transcribed using the Superscript III RT and RNaseOUT kit (Invitrogen Life Technologies, Paisley, UK), as previously reported [17].

### 2.4. Statistical Analysis

#### 2.4.1. Sample Size

Our sample size was calculated on the basis of the results reported by Nabulsi et al. [13], who reported a DAA treatment failure rate of 13.9% in patients with HCV. In total, our study required 13 patients to identify factors that affected treatment outcome on day 13 with an 80% power at a significance level of 5%.

#### 2.4.2. Odds Ratios–Based Approach

The significance of odd ratio (OR) was examined based on *z*-test at the confidence interval (CI) of 95%, i.e., significance level (*P*) of 0.05 (*P* ≤ 0.05), which measures if the OR is statistically significant. This is used in order to show whether or not a given factor or factors have caused the failure to achieve SVR at the 12th week of DAA treatment.

#### 2.4.3. Multiple Logistic Regression Model Development

ORs were used to compare the relative odds of the occurrence of the outcome of HCV treatment, given exposure to the variables. In addition to examining the ORs for each independent variable, we developed the logistic regression model to consider all independent variables concurrently for their correlation with the risk of treatment failure.

In our logistic regression model, the dependent variable was presented in binary form, and independent variables were presented in continuous or categorical form. The dependent variable *Y* has two possible values: *y* = 1 (person does not achieve SVR) and *y* = 0 (person achieves SVR). There were 17 independent variables (*xj*, *j* = 1, ⋯17) (Table 1).

We applied a stepwise logistic regression: First, we considered all 17 independent variables, then developed a smaller model using stepwise regression (add and remove the variable at 0.1 level of significance), and finally examined the smaller model and rebuilt it to include only significant variables. The receiver operating characteristic (ROC) curve (AUC_ROC_) was applied to compare the predictive performance across models using a validation set. Logistic regression was performed using R (version 11) [18].

### 2.5. Ethical Considerations

The study protocols were reviewed and approved by the Institutional Review Board (IRB) of Hai Phong International Hospital, Vietnam (IRB. 20.302). The study was conducted in accordance with the Declaration of Helsinki and the International Conference on the Harmonization of the Technical Requirements for the Registration of Pharmaceuticals for Human Use - Good Clinical Practice guidelines. Prior to data collection, ethical approval was obtained from the ethics subcommittees of Hai Phong International Hospital. All patients provided written informed consent prior to the commencement of the study.

## 3. Results

After 12 weeks of treatment with DAAs, 334 HCV patients were classified into two groups: those who achieved SVR (group 0, *n* = 239) and those who did not achieve SVR (group 1, *n* = 95). Thus, the rate of reaching the treatment goal in our study group was 71.56%. The mean age of group 0 and group 1 was similar (50.72 ± 12.68 vs. 51.23 ± 10.66) with *P* value = 0.732. The proportion of men in groups 0 and 1was 65.69% and 71.58%, respectively (*P* = 0.366). Our study group also evaluated the comorbidities of 334 patients with HCV, including type 2 diabetes, hypertension, peptic ulcer, HBV coinfection, and cancer (excluding liver cancer). We found that the difference in the incidence of these diseases (except for the HBV comorbidity variable) did not differ significantly between groups (*P* > 0.05) (Table 1).

When comparing the clinical and subclinical characteristics between groups 0 and 1, we found many statistically significant differences (Table 1). The rate of cirrhosis in group 0 was 8.79%, while it was 55.79% in group 1 with ORs (95%CI) = 12.91 (7.15, 24.09) and *P* < 0.001. When looking at the SVR rate at week four of DAAs, we found that the SVR rate in group 0 was significantly higher than that in group 1 (49.37% vs. 5.26%) with ORs (95%CI) = 6.71 (3.82, 11.98) and *P* < 0.001. The FIB-4 index before treatment in group 1 was also higher than that of group 0 (mean + SD; 7.39 ± 4.54 vs. 2.25 ± 1.37, respectively, *P* < 0.001). Similarly, pretreatment blood AST (U/l) levels of the 95 patients in group 1 were significantly higher than that of the 239 patients in group 0 (246.88 ± 320.54 vs. 57.35 ± 34.08, sequentially, *P* < 0.001). Group 1 had a statistically significant higher serum ALP level (U/l) before treatment than group 0 (319.42 ± 403.27 vs. 63.6 ± 45.55, respectively, *P* < 0.001). Meanwhile, the mean total bilirubin levels of groups 0 and 1 were similar (14.43 ± 25.81 vs. 17.22 ± 24.68, respectively, *P* = 0.368). We found that log_10_ of hepatitis C viral load before treatment was higher in group 1 than in group 2 (5.04 ± 1.38 vs. 4.14 ± 1.25, *P* < 0.001).

When comparing renal function, we found that the serum creatinine concentration before treatment in both groups was similar (74.58 ± 23.83 vs. 82.68 ± 79.18%, *P* = 0.154). Similarly, the eGFR (mL/min) of HCV patients in group 0 did not differ from that in group 1 (88.24 ± 36.4 vs. 87.7 ± 33.58, *P* = 0.896). The hemoglobin concentration in group 0 was significantly higher than that of the patients in group 1 (138.85 ± 19.65 vs. 123.63 ± 20.33, respectively, *P* < 0.001).

We found that the genotype HCV-1 rate was higher in group 1 (77.89% vs. 20.08%) with ORs (95%CI) = 13.82 (7.86, 25.2) and *P* < 0.001. By contrast, the distribution of HCV-6 genotype was found to be lower in group 1 than in group 0 (7.37% vs. 62.34%, respectively) with ORs (95%CI) = 0.06 (0.02, 0.12) and *P* < 0.001. Meanwhile, the distribution of HCV-2 and HCV-3 genotypes did not differ significantly between the two groups (*P* > 0.05).

### 3.1. Logistic Regression and Interpretation

Firstly, we took into account all 17 independent variables in the full logistic regression model (Table 1). The models with coefficients, standard errors, *P* value, and OR (with 97.5% CI) are given in Table 2. It should be noted that positive coefficients (OR> 1) show that the variable positively relates to the probability of failure to achieve the SVR after 12 weeks of DAA treatment and negative coefficients (OR< 1) suggest a negative association with the probability of treatment failure. However, it is important to observe the significance of independent variables.

As per multiple logistic regression analysis, the independent variables (cirrhosis, HCV genotype, levels of AST, ALP, hemoglobin, fibrosis-4 score, and log_10_ of viral load before treatment) showed significant ORs (Table 1), while other variables (number of comorbidities, count of platelet numbers) did not exhibit significant ORs.

**Figure 1:**
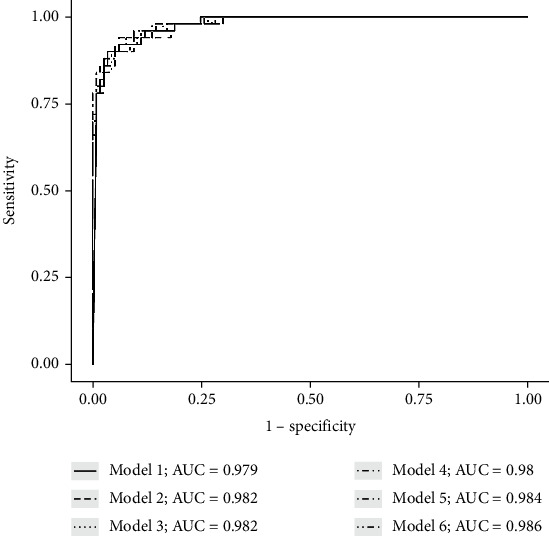
Receiver operating characteristic curves comparing the performance of six regression models using test datasets.

Secondly, we performed stepwise logistic regression to reduce the complete model by removing insignificant variables determined by the preceding stepwise regression. The comprehensive model (model 1) yielded a BIC of 114 with a pseudo *R*^2^ of 0.89. The area under the ROC curve (95% CI) was 0.979 (0.961-0.997). From model 1, we excluded the pretreatment platelet count variable, because it had a *P* value of 0.225, to give model 2 (BIC of 110.6, pseudo *R*^2^ = 0.88). We then removed the variable associated with HBV, because of the *P* value = 0.206, to give model 3 (BIC of 109.2). We further removed the pretreatment eGFR (mL/min) to obtain model 4 (BIC of 102.2). Due to the relationship between eGFR and blood creatinine concentration, we removed the variable creatinine concentration from model 4, to give model 5 (BIC of 101.1) with 12 variables. Model 6 was generated by the removal of the DAA therapy variable from model 5, which did not alter the BIC. A comparison of the statistical differences and fit of the models is presented in Table 3. Model 6 was chosen because of the fewest variables, highest ROC, and lowest BIC. It showed excellent diagnostic performance in predicting HCV treatment failure with a ROC curve (95%CI) = 0.986 (0.971-0.999) (Figure 1).

By running model 6 on the full dataset, we found that the most important predictor associated with failure to reach SVR was achieving RVR at the 4^th^ week (ORs (95%CI) = 51.54 (6.39, 139.82) and p-value = 0.002) followed by the patient's cirrhosis status before treatment (OR, 97.5% =9.72 (2.85, 39.28), *P* = 0.001). Hepatitis C viral load before treatment also played an important role in predicting the risk of HCV treatment failure, ORs (97.5%) = 2.4 (1.52, 4.17), *P* = 0.001. With an increase of 1 in the FIB-4 score, the patient's risk of failure to achieve SVR at 12 weeks increased by 1.83 times (ORs (97.5%) = 1.83 (1.36, 2.67), *P* = 0.001). With 1 U/L higher AST and ALP concentrations than pre-HCV, the patient had a 1.03-and 1.02-fold increased risk of treatment failure, respectively. In contrast, a lower pretreatment hemoglobin level was a risk factor for HCV treatment failure at week 12 with ORs (97.5%) = 0.96 (0.93.0.99), *P* = 0.003. The genotype of the hepatitis C virus has a different influence on the risk of treatment failure. Compared with the HCV-1 genotype, patients with the HCV-6 genotype had a lower risk of failure to achieve SVR at 12 weeks (OR, 97.5%) = 0.32 (0.06.0.87), *P* = 0.053.

## 4. Discussion

Our study assessed the effectiveness of treatment and predicted the risk of failure to achieve SVR by developing a multivariable logistic model of 334 HCV patients. Cure rates of more than 90% have been documented in most phase III clinical trials using DAA therapy in chronic hepatitis C patients. Our study showed that the SVR measured 12 weeks after treatment was only 71.56%. SVR at 12 weeks was considered the primary outcome measure of HCV treatment efficacy by DAA therapy. The new DAA therapy is much more effective than the traditional treatment, which uses peg-IFN with or without protease inhibitor drugs [19]. However, this result is highly dependent on the HCV genotype and absence of cirrhosis [19]. Our study population consisted of 22.16% of patients with cirrhosis, which may have affected the percentage of SVR achievement. Furthermore, considering the success rate by genotype, patients with the HCV-6 genotype had a success rate of nearly 96%, while patients with the HCV-1 genotype had the lowest success rate (35%) (Table 1). Similar to our study, SVR rates of HCV genotype 6 with a combination of pegylated interferon and ribavirin is superior to genotype 1, and nearly comparable to genotype 3, with 60%-90% [20].

RVR is the most important predictor of SVR, as confirmed by logistic regression analysis [21]. In patients with RVR at week 4, SVR at week 24 was 81.6%, while in patients without RVR, the SVR rate was 52.1%, if the treatment duration was 24 weeks. Our study showed that in the group of patients achieving RVR, the SVR at week 12 was 49.37%, while in the group not achieving RVR, the SVR rate was very low at 5.26% (Table 1). An observational cohort of 21,142 HCV patients with genotype-1 initiating 8 or 12 weeks of DAA therapy also reported that detectable 4-week on-treatment HCV RNA ≥ 15 IU/ml reduced SVR odds ratios [22].

Hepatitis C patients with cirrhosis are considered difficult to treat and achieve lower SVR rates than those without cirrhosis. The efficacy of telaprevir or boceprevir in treatment-experienced patients with HCV genotype 1 and cirrhosis was studied in the CUPIC study [23]. A relatively high proportion of patients with HCV genotype 1 infection and cirrhosis respond to the combination of peginterferon and ribavirin plus telaprevir or boceprevir. However, side effects are frequent and often serious. Therefore, treatment with first-generation DAAs is not recommended for patients with advanced cirrhosis and severe portal hypertension. Our study demonstrated that cirrhosis was also a risk factor for treatment failure at week 12 of DAA therapy. About half (53/95) of the patients who did not achieve SVR12 had cirrhosis. One-way ANOVA showed that the risk of failure to achieve SVR12 was 12.91 times higher in the cirrhotic group than in the non-cirrhotic group with OR (95% CI) =12.91 (7.15-24.09), *P* = 0.001. Our study found no effect of renal function on the likelihood of achieving SVR at the 12^th^ week of DAA treatment. In a review study evaluating the effectiveness and safety of DAA in the treatment of patients with chronic kidney disease, the authors concluded that the treatment of chronic hepatitis C in patients with chronic kidney disease was highly effective with SVR12 rates similar to those seen in patients without CKD and with acceptable adverse events [24]. Our study found that AST and ALP levels were associated with the risk of failure to achieve SVR at week 12 of DAA therapy (Tables 1 and 3). A prospective study based on demographic and clinical data was obtained from 834 patients with SVR after HCV treatment with PegIFN- or DAA-based regimens. Advanced liver disease and markers of hepatic steatosis were confirmed by the team to be independently associated with elevated aminotransferase levels after SVR [25].

FIB-4 is a simple, easy-to-calculate, and practical tool that includes patient age, ALT levels, AST levels, and platelet counts, developed and validated as a noninvasive marker for liver fibrosis. The FIB-4 score has also been utilized by multiple studies to correlate hepatocellular carcinoma risk in DAA-treated non-cirrhotic HCV patients and in patients with chronic HBV, non-alcoholic fatty liver disease, or alcoholic cirrhosis [26, 27]. An increased FIB-4 score (≥3.25) has a high specificity (>96%) for advanced liver fibrosis, while a low FIB-4 score (≤1.45) is related to low fibrosis stages. Our study found that the FIB-4 score was closely related to the rate of treatment failure. A retrospective study of 363 chronic hepatitis C patients who completed a course of DAA at three hepatitis clinics in Spain concluded that advanced liver fibrosis and HIV coinfection were significantly associated with treatment failure [28]. Similarly, a retrospective cohort analysis of 784 people living with human immunodeficiency virus reported advanced liver fibrosis as a predictor of HCV viral failure with an OR of 2.29 [29].

In the multivariate logistic model with the entire dataset (Table 4), we included hemoglobin level as one of the predictors with OR (95% CI) = 0.96 (0.93, 0.99), *P* *value* = 0.003. In a retrospective study of 152 HCV patients receiving ribavirin +DAA, 15.1% experienced anemia. Baseline serum Hb and Hb% drop was a significant predictor of the risk of anemia [30]. The effect of anemia on the efficacy and safety of DAA therapies for patients with chronic kidney disease was evaluated in a single-center retrospective study. The author group indicated that Hb levels of <10.5 g/dL prior to DAA treatment did not affect the virological response in renal patients but were related to augmented serum creatinine [31].

Our study found hepatitis C virus genotype to be one of the predictors of treatment failure at week 12. Four genotypes were detected among 334 hepatitis C patients of our study including HCV-1, HCV-2, HCV-3, and HCV-6; multivariate logistic regression analysis showed that patients with HCV-6 had a lower risk of failure to achieve SVR12 when compared with those with genotype 1. Genotypes 1, 2, and 3 of the hepatitis C virus are widely distributed worldwide, making them the focus of *in vitro* studies and clinical trials [32]. In contrast, genotypes 4 and 5 were discovered mainly in African and Middle Eastern countries. Interestingly, genotype 6 of hepatitis C virus is found mainly in Southeast Asian countries, including Vietnam [7, 33]. A review aimed at summarizing data regarding HCV genotype 6 treatment found that genotype 6 HCV treatment outcomes with a combination of pegylated interferon and ribavirin were superior to that of genotype 1 [20]. This study also concluded that patients with RVR are suitable for a 24-week treatment with expected SVR rates of >80%. A study of available SVR12 data from 13951 patients at the Taiwan HCV Registry program showed that genotype-3 had adjusted OR (95% CI) = 5.78 (2.25-14), *P* = 0.0003, compared to genotype-1, while HCV genotype-2 had adjusted OR (95% CI) =1.55 (1.05-2.29), *P* = 0.03 [34]. In our study, multivariate analysis also showed that patients with genotype 2 had higher risk than those with genotype 1 with ORs (95% CI) =12.44 (0.91, 293.43) (Table 2). However, the *P* value was not statistically significant (*P* = 0.08).

A model developed from an observational retrospective cohort study after 12 weeks of DAA regimen determined the predictors of treatment failure including age, history of HCC, insurance type, HIV coinfection, current use of opioid substitution therapy, HCV treatment history, history of solid organ transplantation, current cirrhosis, and history of alcohol use [13]. Our model included specific laboratory data that can directly evaluate the liver and renal functions, which can reduce the errors due to the subjective assessment of physicians. We also included the relationship between anemia through serum hemoglobin level and HCV treatment failure in a multiple regression model.

Our modelling did not handle continuous input variables by dividing the subjects into categories. Keeping the quantitative data constant made it easy for us to integrate the model into electronic medical records to help doctors predict after having input data entered by nurses or laboratories. Additionally, our model, derived from a specific set of outpatients, may not apply to inpatients with different characteristics. Polymorphisms located near the gene encoding the interferon-lambda beta subunit (IL28B) can identify patients with a high probability of achieving SVR [12]. In our study, the polymorphism of IL28B was not considered, as this factor mainly predicts efficacy in patients receiving ribavirin therapy. A monotone likelihood limitation existed in our clinical data, in which the odds ratio of genotype 1 (Table 1), RVR (Table 2), and cirrhosis (Table 4) inflated due to the small number of samples with substantial censoring of survival time and several highly predictive covariates [35]. Nevertheless, our logistic regression model demonstrated the role of predictors in estimating the risk of treatment failure in patients with HCV.

## 5. Conclusions

Our study followed up 334 patients divided into two groups according to their achievement of SVR. Univariate analysis showed that SVR failure rate was influenced by cirrhosis status, HBV comorbidity, RVR achievement at week 4, genotype, FIB-4 score, AST level, ALT level, serum hemoglobin levels, and hepatitis C viral load before treatment. An excellent predictive performance of the multiple logistic regression model was built with AUC_ROC_ (95% CI) =0.986 (0.971-0.999) that can help clinicians select optimal treatment strategies and issue the most accurate disease prognosis for patients with chronic hepatitis C.

## Figures and Tables

**Table 1:** Comparison of clinical characteristics between group 0 (*n* = 239) and group 1 (*n* = 95).

Characteristic	Group 0 (*n* = 239)	Group 1 (*n* = 95)	*R* ^2^	OR (95% CI)	*P* value
Age, years, mean ± SD	50.72 ± 12.68	51.23 ± 10.65	0.01		0.732
Sex (male), *n* (%)	157 (65.69)	68 (71.58)		1.32 (0.79, 2.24)	0.366
BMI, mean ± SD	0.19 ± 0.03	0.19 ± 0.03	0.01		0.686
Cirrhosis (Y/N), *n* (%)	21 (8.79)	53 (55.79)		12.91 (7.15, 24.09)	0.001
Comorbidity					
Diabetes, *n* (%)	24 (10.04)	7 (7.37)		0.73 (0.28, 1.68)	0.535
Hypertension, *n* (%)	14 (5.86)	8 (8.42)		1.49 (0.57, 3.64)	0.464
Ulcer, *n* (%)	22 (9.21)	16 (16.84)		2 (0.98, 4)	0.057
HBV-HCV coinfection, *n* (%)	5 (2.09)	55 (57.89)		61.49 (25.24, 187.94)	0.001
Malignancy (not including HCC)	8 (3.35)	3 (3.16)		0.98 (0.2, 3.53)	1
Drug regimen					
SOF/LDV, *n* (%)	155 (64.85)	66 (69.47)		1.24 (0.75, 2.08)	0.445
SOF/VEL, *n* (%)	83 (34.73)	30 (31.58)		0.87 (0.52, 1.44)	0.611
Reach RVR, *n* (%)	118 (49.37)	5 (5.26)		0.06 (0.02, 0.14)	0.001
FIB-4 score, mean ± SD	2.25 ± 1.36	7.39 ± 4.54	0.43		0.001
Baseline AST (U/L), mean ± SD	57.35 ± 34.07	246.88 ± 320.53	0.20		0.001
Baseline ALP (U/L), mean ± SD	63.6 ± 45.55	319.42 ± 403.26	0.22		0.001
Baseline total bilirubin (mg/dl), mean ± SD	14.43 ± 25.81	17.22 ± 24.67	0.01		0.368
Baseline serum creatinine, (*μ*mol/L), mean ± SD	74.58 ± 23.82	82.68 ± 79.18	0.01		0.154
Baseline GFR (mL/min), mean ± SD	87.7 ± 33.57	88.24 ± 36.39	0.01		0.896
Baseline hemoglobin (g/L), mean ± SD	138.85 ± 19.65	123.63 ± 20.32	0.11		0.001
Baseline viral load log_10_ IU/ml, mean ± SD	4.14 ± 1.25	5.04 ± 1.37	0.10		0.001
Viral C genotype					
Genotype 1, *n* (%)	48 (20.08)	74 (77.89)		13.82 (7.86, 25.2)	0.001
Genotype 2, *n* (%)	25 (10.46)	9 (9.47)		0.91 (0.39, 1.97)	1
Genotype 3, *n* (%)	17 (7.11)	5 (5.26)		0.75 (0.24, 1.96)	0.632
Genotype 6, *n* (%)	149 (62.34)	7 (7.37)		0.05 (0.02, 0.11)	0.001

BMI, body mass index; HBV, hepatitis B virus; HCV, hepatitis C virus; RVR, rapid virological response; LDV/SOF, ledipasvir/sofosbuvir; VEL/SOF: sofosbuvir/velpatasvir; AST: aspartate aminotransferase; ALT: alanine transaminase; FIB-4: fibrosis index based on four factors; GFR: glomerular filtration rate.

**Table 2:** Multiple variable logistic regression models based on the test dataset.

Model	Independent variable	Slope	SE	*P*	OR (97.5%CI)
Model 1	Intercept	-55.38	33.69	0.101	0.01 (0.01, 0.01)
	Number of comorbidities	-2.06	1.56	0.186	0.13 (0.01, 1.5)
	Cirrhosis	10.59	4.75	0.026	39344.68 (37.88, 4887573)
	HBV coinfection	8.01	5.88	0.174	3004.94 (0.49, 1197994)
	Genotype 2	8.18	4.39	0.063	3564.85 (6.24, 4097476)
	Genotype 3	-2.99	2.41	0.214	0.06 (0.01, 3.83)
	Genotype 6	-2.17	1.88	0.249	0.12 (0.01, 3.52)
	Medication (SOF.VEL)	6.06	2.86	0.034	428.1 (5.32, 717602.2)
	Baseline AST	0.14	0.08	0.057	1.15 (1.05, 1.44)
	Baseline ALP	0.12	0.07	0.069	1.12 (1.03, 1.36)
	Baseline creatinine	0.19	0.1	0.056	1.2 (1.05, 1.59)
	Baseline GFR	0.1	0.06	0.09	1.1 (1.01, 1.29)
	Baseline hemoglobin	-0.35	0.18	0.053	0.71 (0.41, 0.9)
	Plt_pre	0.03	0.02	0.225	1.03 (1, 1.08)
	FIB_4	3.48	1.66	0.036	32.45 (3.27, 4953.69)
	Baseline viral load (log10 IU/ml)	3.03	1.58	0.056	20.52 (2.71, 3935)
	RVR	22.94	24.65	0.353	91353 (250.95, 1024875)

Model 2	Intercept	-44.75	18.76	0.017	0.01 (0.01, 0.01)
	Number of comorbidities	-2.22	1.4	0.111	0.11 (0.01, 1.05)
	Cirrhosis	10.43	4.11	0.012	33528.71 (52.01, 1364237)
	HBV coinfection	6.21	4.9	0.206	494.86 (0.21, 411352200)
	Genotype 2	7.17	3.72	0.054	1296.44 (4.39,84435790)
	Genotype 3	-3.06	2.14	0.153	0.05 (0.01, 2.44)
	Genotype 6	-2.71	1.79	0.129	0.07 (0.01, 1.67)
	Medication SOF.VEL	6.89	2.99	0.022	975.47 (9.17, 2460177)
	Baseline AST	0.13	0.06	0.033	1.14 (1.05, 1.33)
	Baseline ALP	0.1	0.05	0.047	1.1 (1.03, 1.24)
	Baseline creatinine	0.15	0.08	0.036	1.16 (1.05, 1.41)
	Baseline GFR	0.09	0.05	0.063	1.09 (1.01, 1.23)
	Baseline hemoglobin	-0.31	0.14	0.024	0.74 (0.51, 0.9)
	FIB_4	3.31	1.35	0.015	27.17 (3.64, 1049.77)
	Baseline viral load (log10 IU/ml)	2.69	1.26	0.033	14.64 (2.41, 517.5)
	RVR	21.39	9.28	0.022	1933120000 (4542.83, 7.439031E+21)

Model 3	Intercept	-30.52	11.36	0.008	0.01 (0.01, 0.01)
	Cirrhosis	7.23	2.82	0.011	1372.69 (16.17, 1459831)
	HBV coinfection	0.75	2.35	0.75	2.12 (0.03, 642.88)
	Genotype 2	5.8	2.64	0.028	328.87 (4.3, 236966)
	Genotype 3	-2.18	2.05	0.289	0.12 (0.01, 3.62)
	Genotype 6	-2.3	1.63	0.157	0.11 (0.01, 1.85)
	Medication SOF.VEL	4.31	2.02	0.033	73.79 (3.02, 17293.09)
	Baseline AST	0.08	0.03	0.012	1.08 (1.03, 1.17)
	Baseline ALP	0.04	0.03	0.052	1.05 (1.01, 1.1)
	Baseline creatinine	0.1	0.05	0.033	1.1 (1.03, 1.23)
	Baseline GFR	0.06	0.04	0.106	1.06 (1, 1.15)
	Baseline hemoglobin	-0.18	0.08	0.019	0.84 (0.68, 0.94)
	FIB_4	2.11	0.78	0.007	8.18 (2.46, 60.01)
	Baseline viral load (log10 IU/ml)	1.51	0.66	0.022	4.51 (1.7, 26.08)
	RVR	14.94	5.58	0.008	3058150 (791.41, 27932120000000)

Model 4	Intercept	-20.77	7.95	0.009	0.01 (0.01, 0.01)
	Cirrhosis	4.9	1.87	0.009	134.18 (7.16, 16212.11)
	Genotype 2	3.43	1.93	0.076	30.85 (1.38, 5070.96)
	Genotype 3	-2.44	1.95	0.212	0.09 (0.01, 2.33)
	Genotype 6	-2.97	1.55	0.056	0.06 (0.01, 0.76)
	Medication SOF.VEL	4.03	2.17	0.063	55.74 (2.1, 14922.96)
	Baseline AST	0.06	0.03	0.012	1.07 (1.03, 1.13)
	Baseline ALP	0.03	0.02	0.067	1.03 (1.01, 1.08)
	Baseline creatinine	0.06	0.04	0.08	1.06 (1.01, 1.15)
	Baseline hemoglobin	-0.13	0.07	0.041	0.89 (0.76, 0.97)
	FIB_4	1.47	0.56	0.008	4.34 (1.87, 18.26)
	Baseline viral load (log10 IU/ml)	1.29	0.53	0.014	3.63 (1.56, 14.42)
	RVR	12.04	4.6	0.009	169281.6 (211.77, 35097410000)

Model 5	Intercept	-16.86	6.76	0.013	0.01 (0.01, 0.01)
	Cirrhosis	4.05	1.51	0.008	57.3 (4.77, 2162.94)
	Genotype 2	2.79	1.62	0.085	16.26 (1.01, 819.57)
	Genotype 3	-2.1	1.71	0.221	0.13 (0.01, 2.36)
	Genotype 6	-2.83	1.53	0.064	0.06 (0.01, 0.83)
	Medication SOF.VEL	3.13	1.68	0.062	22.73 (1.45, 1374.49)
	Baseline AST	0.06	0.02	0.009	1.06 (1.02, 1.11)
	Baseline ALP	0.03	0.02	0.054	1.03 (1.01, 1.08)
	Baseline hemoglobin	-0.1	0.05	0.04	0.92 (0.82, 0.98)
	FIB_4	1.28	0.47	0.006	3.58 (1.72, 11.6)
	Baseline viral load (log10 IU/ml)	1.34	0.49	0.006	3.81 (1.73, 13.37)
	RVR	9.26	3.37	0.007	10411.78 (60.84, 45946530)

Model 6	Intercept	-11.61	5.13	0.024	0.01 (0.01, 0.1)
	Cirrhosis	3.74	1.3	0.004	41.71 (4.65, 893.62)
	Genotype 2	2.53	1.44	0.08	12.44 (0.91, 293.43)
	Genotype 3	-1.43	1.32	0.279	0.25 (0.02, 2.85)
	Genotype 6	-2.94	1.48	0.048	0.06 (0.01, 0.7)
	Baseline AST	0.04	0.02	0.01	1.04 (1.02, 1.08)
	Baseline ALP	0.03	0.01	0.037	1.03 (1.01, 1.05)
	Baseline hemoglobin	-0.07	0.04	0.027	0.94 (0.87, 0.99)
	FIB_4	0.91	0.34	0.007	2.47 (1.4, 5.33)
	Baseline viral load (log10 IU/ml)	1.03	0.39	0.008	2.8 (1.46, 6.9)
	RVR	6.06	2.11	0.004	427.63 (15.54, 7179.67)

HBV, hepatitis B virus; HCV, hepatitis C virus; RVR, rapid virological response; LDV/SOF, ledipasvir/sofosbuvir; VEL/SOF: sofosbuvir/velpatasvir; AST: aspartate aminotransferase; ALT: alanine transaminase; FIB-4: fibrosis index based on four factors; GFR: glomerular filtration rate.

**Table 3:** Comparison of clinical models based on six machine learning classifiers in predicting therapeutic failure among patients with HCV.

Model	No of variables	AUC_ROC_	95% CI (Delong)	Pseudo *R*^2^	BIC	*P*_value (>chi)
Model 1	17	0.979	0.961-0.997	0.89	114	
Model 2	16	0.982	0.965-0.998	0.88	110.6	0.18224
Model 3	15	0.982	0.965-0.997	0.86	109.2	0.05621
Model 4	13	0.98	0.962-0.996	0.84	102.2	0.19101
Model 5	12	0.984	0.968-0.998	0.82	101.1	0.04548
Model 6	11	0.986	0.971-0.999	0.8	101.1	0.02378

AUC_ROC_: area under the receiver operating characteristic curve; BIC: Bayesian Information Criterion.

**Table 4:** Regression coefficients and odds ratios (and 95% CIs) for risk factors associated with treatment failure after 12 weeks of DAA therapy in HCV patients (*n* = 334).

Independent variable	Slope	SE	*P*	OR (95% CI)
Intercept	-8.80	2.84	0.002	0.01 (0.01, 0.03)
Cirrhosis	2.28	0.67	0.001	9.72 (2.85, 39.28)
Genotype 2	0.96	0.85	0.262	2.6 (0.49, 14.29)
Genotype 3	-0.56	0.96	0.559	0.58 (0.09, 3.81)
Genotype 6	-1.17	0.82	0.053	0.32 (0.06, 0.87)
Baseline AST	0.03	0.01	0.003	1.03 (1.02, 1.05)
Baseline ALP	0.02	0.01	0.037	1.02 (1.01, 1.03)
Baseline hemoglobin	-0.05	0.02	0.003	0.96 (0.93, 0.99)
FIB-4	0.61	0.18	0.001	1.83 (1.36, 2.67)
Baseline viral load (log10 IU/ml)	0.88	0.26	0.001	2.4 (1.52, 4.17)
RVR	3.95	1.25	0.002	51.54 (6.39, 139.82)

RVR, rapid virological response; AST: aspartate aminotransferase; ALT: alanine transaminase; FIB-4: fibrosis index based on four factors.

## Data Availability

Data is available on request from the authors.
